# Psychiatric Illness and Intellectual Disability in the Prader–Willi Syndrome with Different Molecular Defects - A Meta Analysis

**DOI:** 10.1371/journal.pone.0072640

**Published:** 2013-08-14

**Authors:** Lin Yang, Guo-dong Zhan, Jun-jie Ding, Hui-jun Wang, Duan Ma, Guo-ying Huang, Wen-hao Zhou

**Affiliations:** 1 Children’s Hospital, Fudan University, Shanghai, China; 2 Key Laboratory of Molecular Medicine, Ministry of Education, Fudan University, Shanghai, China; Institut Jacques Monod, France

## Abstract

**Background and Objectives:**

Several studies have suggested a difference in clinical features of intellectual ability and psychiatric illness in the Prader–Willi syndrome (PWS) with the 15q11-q13 paternal deletion and maternal uniparental disomy (mUPD). Our objective was to appraise evidence on this association through a meta-analysis.

**Methods:**

The electronic records PubMed and EMBASE from 1956 to 2012 were extracted for meta-analysis. Meta-analyses were performed by using fixed effect model. Mean difference, odds ratio, and 95% confidence interval were calculated.

**Results:**

We retrieved a total of 744 PWS cases from 13 studies. These include 423 cases with paternal 15q11-q13 deletions and 318 cases of mUPD. Compare to the PWS cases with mUPD, PWS patients with the paternal 15q11-q13 deletion associated with significantly lower full scale IQ (FSIQ) [mean difference (MD), -2.69; 95%CI, -4.86 to -0.52; *p*=0.02] and verbal IQ (VIQ) (MD, -7.5; 95%CI, -9.75 to -5.26; *p*<0.00001) but higher performance IQ (PIQ) (MD, 4.02; 95%CI, 1.13 to 6.91; *p*=0.006). In contrast, PWS patients with mUPD are associated with significantly higher risk of psychiatric illness [odds rate (OR), 0.14; 95%CI, 0.08 to 0.23; *p*<0.00001] and higher risk of bipolar disorder (OR, 0.04; 95%CI, 0.01 to 0.23; *p*=0.0002).

**Conclusions:**

Significant different clinical features of cognitive development and psychiatric illness are associated with PWS with different molecular defects. These findings provide support for evidence based practice to evaluate and manage the PWS syndrome with different molecular defects.

## Introduction

Prader–Willi syndrome (PWS) is a genomic imprinting disorder caused by deficiency of paternally expressed gene or genes in the chromosome 15q11-13 region [1–4]. Most patients are caused by a paternal deletion on the chromosome 15q11–q13 (70%-75%) and a maternal uniparental disomy (mUPD) of chromosome 15 (25%-29%). The imprinting center defect is found in less than 1% of PWS cases. The estimated prevalence of PWS is 1:10,000 to 1:30,000 [5–7]. The clinical features of PWS include neonatal hypotonia, feeding difficulty, respiratory problem at birth, hypogonadism, cognitive disabilities, hyperphagia, childhood onset obesity, obsessive and compulsive behaviors, and psychiatric illness in adult [8,9]. The diagnostic criteria for the Prader–Willi syndrome (PWS) developed in 1993 have proven to be a useful tool in clinic setting [10]. However, the confirmation of clinical diagnosis usually relies on the molecular genetic testing.

Although the majority of clinical features of PWS are well recognized and characterized, the behavioral features associated with PWS has not been fully investigated. The number of new PWS cases, caused by either paternal deletion of 15q11-q13 or mUPD, has been increased significantly in recent years due to available array CGH (comparative genomic hybridization) technique in clinical diagnostic laboratory. The different clinical presentations related to behaviors and mental health have been suggested between individuals with paternal deletion of 15q11-q13 and mUPD [11–13]. These differences appear more prominent in the domains of the neurodevelopment and psychiatric presentations. Individuals with mUPD are more likely to have psychosis [14,15], autism spectrum disorders [16–19], and milder intellectual disability than other causes [20–22]. Individuals with paternal deletion of 15q11-q13 showed a higher frequency of impaired speech articulation and language development [23].

There is, however, no consensus as to whether the difference in the neurodevelopment outcomes between 15q11-q13 paternal deletion and mUPD are evident and clinically significantly. This information, however, is important to counsel family and develop anticipatory care. Therefore, we reviewed published literature on this topic systematically and conducted a meta-analysis to evaluate the level of intellectual disability and frequency of psychiatric illness and their correlations between PWS cases caused by the 15q11-q13 paternal deletion and mUPD.

## Methods

### Literature Search

A comprehensive literature search was performed to identify the relevant studies in PubMed, the Cochrane Central Register of Controlled Trials and EMBASE from 1956 to May 31, 2012. Using Medical Subject Headings terms, searches were limited to original studies in human. The keyword of Prader–Willi syndrome was used in combination with deletion or UPD.

### Study Selection and Data Extraction

The Endnote X4 and NoteExpress2 software were used to compile the data for meta-analysis. The PWS cases confirmed molecularly with either paternal deletion or mUPD were included for meta-analysis. We contacted corresponding authors of the studies for additional information as needed. The studies reported more than one of the outcomes of neurodevelopment were included in the metaanalysis: 1) IQ: full scale IQ (FSIQ), verbal IQ (VIQ) and performance IQ (PIQ); 2) psychiatric illness including depressive psychosis, bipolar disorder or other psychotic illness. The studies were excluded for meta-analysis if required data were incomplete. Two reviewers independently screened all studies for those requiring further retrieval (full text or abstract), and then independently reviewed these studies to identify whether they met the inclusion criteria. Disagreements regarding trial eligibility were discussed and resolved by two reviewers.

### Statistical Analysis

We report outcomes using odds rate (OR) with 95% confidence intervals (CIs) for dichotomous data and weighted mean difference (MD) with 95% CI for continuous data. *I* ^2^ statistics were used to measure heterogeneity of the studies. If the *I* ^2^ value was less than 50%, a fixed effects meta-analysis was applied. If the *I* ^2^ value was more than 50%, the random-effects meta-analysis was performed. Potential publication bias was investigated by visual assessment of the funnel plot (plots of effect estimates against sample size). Metaanalyses and funnel plot calculation were performed using Review Manager software version 5.1 (Nordic Cochrane Centre, Copenhagen, Denmark). Statistical significance was determined if the 2-sided P value was less than 0.05.

## Results

### Search Results and Study Characteristics

**Figure 1:**
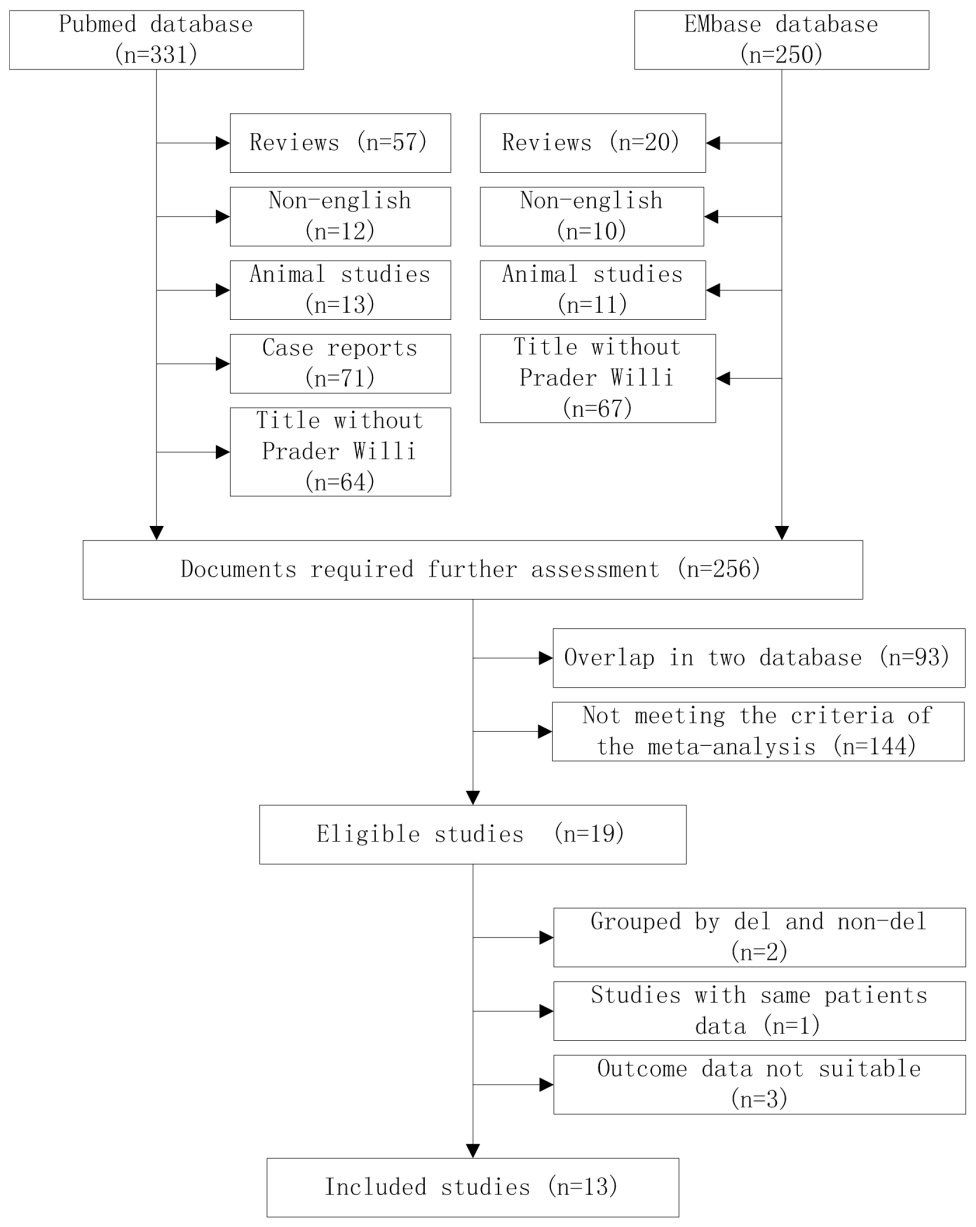
Flow chart of articles screening and selection process.

A total of 581 records were identified from the electronic database, 331 records from PubMed, 250 records from EMBASE. Seventy-seven reviews, 24 animal studies, and 71 single case reports were immediately removed. A total of 22 articles were removed due to language barrier and 131 articles without Prader-willi in their title were removed. Ninety three studies were duplicated in two databases. One hundred fifty studies did not meet the inclusion criteria or because of incomplete data in the papers. After these careful filtering, a total of 13 studies [11,19,21,24–33] met the inclusion criteria of the meta-analyses. Flow chart of articles screening is presented in Figure 1. There are 744 PWS cases in 13 studies. Among them, 423 PWS patients with 15q11-q13 paternal deletion (56.9%), 318 with mUPD (42.7%), 3 with imprinting center defect. The distribution of three molecular defects is consistent with other studies in general but with a slightly higher percentage of mUPD [34]. The characteristics of included studies are available in Table 1. Six studies [11,21,27,30,32,33] were carried out in the USA, four [26,28,29,31] in the UK, two [24,25] in Netherlands, one [19] in Belgium. Of the studies included in the systematic review, 11 studies [11,19,21,26–33] reported IQ score (Table 2), 5 of these studies [21,26,27,29,30] were using the Wechsler Intelligence Scale, 1 study [11] was using the K-BIT scale, 1 study [28] was using Wechsler and Raren’s, 1 study [33] was using Kaufman or Wechsler, and 3 studies [19,31,32] did not provide detail description for tools used. Five studies [11,24–26,31] reported psychiatric illness: 3 studies [24,26,31] used the ICD-10 criteria for the diagnosis and 2 studies [11,25] used psychiatric diagnosis in medical records. The clinical diagnosis in all cases were confirmed by molecular genetic testing using DNA methylation analysis, fluorescence in situ hybridization (FISH), DNA microsatellite analysis or multiplex ligation-dependent probe amplification (MLPA). In 3 studies [11,27,30], the paternal deletions of 15q11-q13 are further divided into class I and class II deletion II based on the genomic locations of breakpoints as previously described [30,35]. Weighted average method was used to combine the IQ scores of the two deletion subgroups.

**Table 1:** Descriptive characteristics of the included studies.

Author	Year	Country of study	Case Number	Male	Deletion	mUPD	Age (y) M+SD (Range)	PWS diagnosis	Outcome
Sinnema et al. [24]	2011	Netherlands	97	NA	53	44	36.2+12.4 (18-66)	MLPA	Psychiatry
Mass et al. [25]	2010	Netherlands	79	34	45	33	34.4+11.8 (18-65)	DNA methylation studies on the SNURF/SNRPN locus	Psychiatry
Dykens et al. [11]	2008	USA	88	43	55	33	22.41+11.74 (5-51)	FISH, methylation studies, or MLPA/MS-MLPA	Psychiatry, IQ
Soni et al. [26]	2008	UK	46	21	24	22	31.2+9.6 (12-51)	microsatellite analysis	Psychiatry, IQ
Zarcone et al. [27]	2007	USA	73	20	42	31	22.7+9.4 (10-40)	FISH and DNA microsatellite analysis	IQ
Descheemaeker et al. [19]	2006	Belgium	59	31	40	18	21.2 (2-51)	chromosome examination and DNA methylation	IQ
Milner et al. [28]	2005	UK	96	51	47	49	16.3+12.4 (3.3-50)	quantitative fluorescent PCR assay, microsatellite analysis	IQ
Walley et al. [29]	2005	UK	18	11	12	6	23.5+7.8 (16-49)	molecular genetic testing, no detail	IQ
Bulter et al. [30]	2004	USA	46	21	25	21	23+8.6	FISH, methylation and microsatellite analysis	IQ
Boer et al. [31]	2002	UK	15	8	9	5	38.5+5.7 (29-47)	methylation pattern	Psychiatry, IQ
FOX et al. [32]	2001	USA	43	NA	24	19	23.3+8.7	FISH, methylation and microsatellite analysis	IQ
Roof et al. [21]	2000	USA	38	16	24	14	22.2+9.1 (10-44)	high-resolution chromosome analysis, DNA microsatellite analysis	IQ
Dykens et al. [33]	1999	USA	46	18	23	23	17+9.42 (6-42)	molecular genetic testing, no detail	IQ

Note: MLPA, multiplex ligation-dependent probe amplification; FISH, fluorescence in situ hybridization; NA: not available.

**Table 2:** Characteristics of the IQ reported studies.

Author	Year	Diagnostic criteria	Perform	FSIQ		VIQ		PIQ	
				DEL (M, SD)	UPD (M, SD)	DEL (M, SD)	UPD (M, SD)	DEL (M, SD)	UPD (M, SD)
Dykens et al.	2008	K-BIT2	NA	62.07, 12.3 (n=55)	64.96, 13.07 (n=33)	-	-	-	-
Soni et al.	2008	Wechsler	carry out by the first author	64.5,8.5 (n=24)	68.7,11.2 (n=22)	-	-	-	-
Zarcone et al.	2007	Wechsler	Trained research staff conducted IQ test	62.3, 55.1 (n=41)	65.2, 12.1 (n=30)	63.3, 9.3 (n=41)	71.3, 11.9 (n=30)	65.7, 9.3 (n=41)	63.1, 11.3 (n=30)
Descheemaeker et al.	2006	NA	NA	57.2, 16.21 (n=40)	58.4, 15.56 (n=18)	-	-	-	-
Milner et al.	2005	Wechsler and Raven’s	the researchers involved were blind to the genetic status of participants.	70.78, 16.21 (n=45)	69.49, 16.89 (n=47)	81.16, 17.4 (n=19)	79.62, 21.08 (n=21)	76.53, 14.43 (n=19)	68.57, 10.44 (n=21)
Walley et al.	2005	Wechsler	NA	-	-	71.64, 12.08 (n=12)	76.50, 8.64 (n=6)	-	-
Bulter et al.	2004	Wechsler	NA	-	-	62.52, 9.18 (n=25)	70, 6.20 (n=21)	-	-
Boer et al.	2002	NA	NA	67.0, 12.39 (n=9)	62.4, 9.86 (n=5)	-	-	-	-
Fox et al.	2001	NA	NA	62.7, 9.8 (n=24)	63.9, 6.4 (n=19)	62.1, 9.5 (n=24)	69.7, 5.5 (n=19)	66.5, 9.6 (n=24)	61.6, 7.7 (n=19)
Roof et al.	2000	Wechsler	carry on by a licensed psychological examiner	61, 9.2 (n=24)	64.1, 7.9 (n=14)	60.8, 8.6 (n=24)	69.9, 6.4 (n=14)	64.7, 9.3 (n=24)	62.2, 9.7 (n=14)
Dykens et al.	1999	Kaufman or Wechsler	two of the university clinics	62.97, 8.83 (n=23)	70.93, 11.15 (n=23)	-	-	-	-

NA: not available

### Results of Meta-analysis

#### FSIQ in the paternal deletion and mUPD subgroup

**Figure 2:**
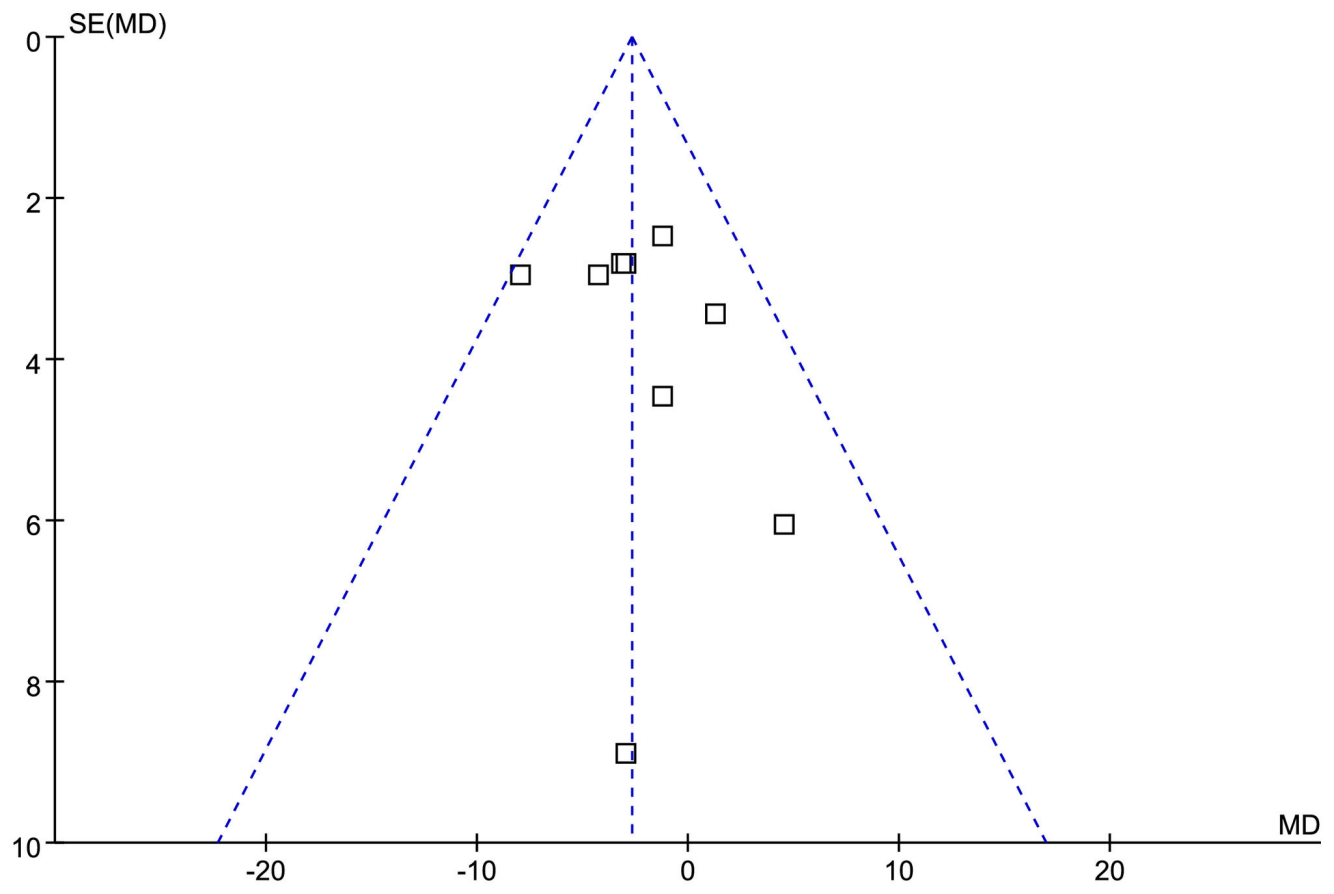
Funnel plot for publication bias about FSIQ.

**Figure 3:**
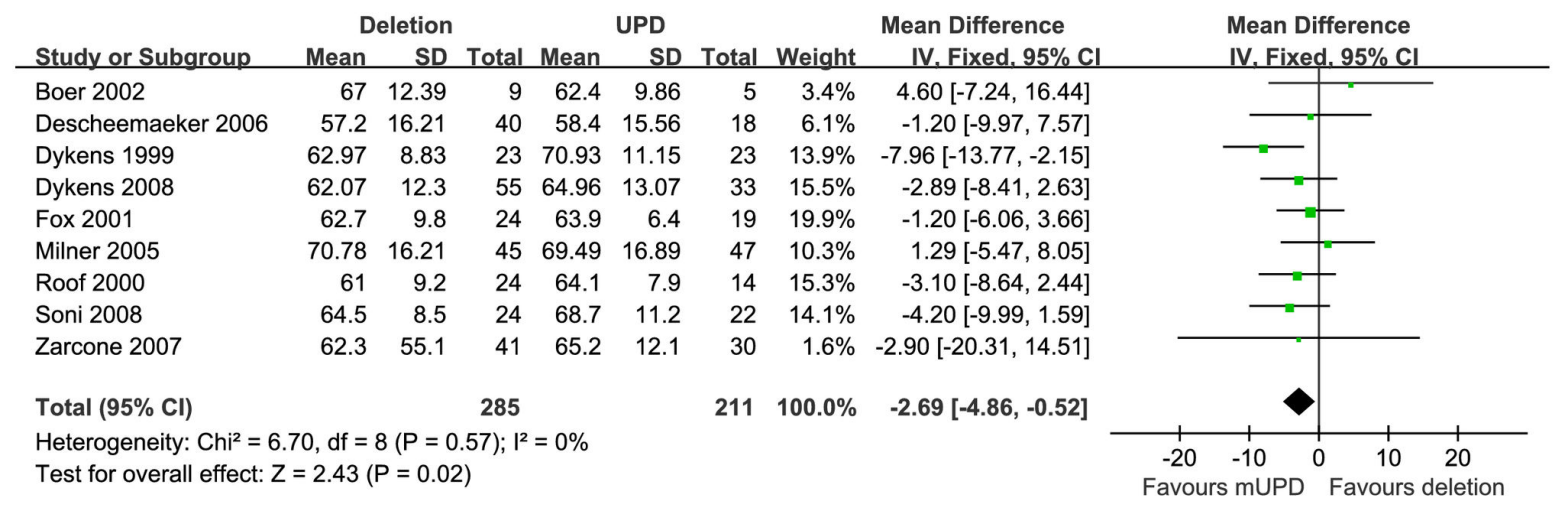
Meta analysis of the FSIQ in DEL and UPD groups.

Funnel plot for publication bias about FSIQ is presented in Figure 2. Nine studies [11,19,21,26–28,31–33] provided FSIQ scores of PWS patients with 15q11-q13 paternal deletion or mUPD. These studies encompassed 285 cases with the paternal deletion and 211 cases with mUPD. Compared with two molecular groups, the PWS cases with deletion have statistically significant lower FSIQ scores than that of mUPD [MD =-2.69 (95% confidence interval (CI), -4.86 to -0.52; *p*=0.02)] and results were homogeneously distributed (*p*=0.57) (Figure 3).

#### VIQ in paternal deletion and mUPD subgroups

**Figure 4:**
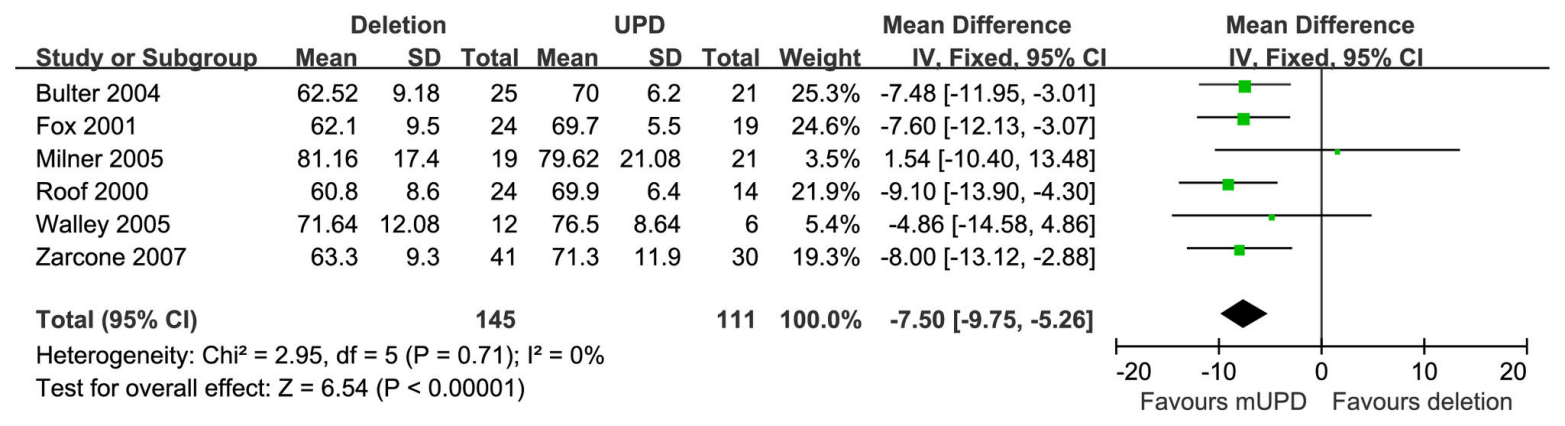
Meta analysis of the VIQ in DEL and UPD groups.

Six studies [21,27–30,32] provided VIQ scores of PWS patients with 15q11-q13 paternal deletion or mUPD. These studies included 145 cases with paternal deletion and 111 cases with mUPD. Compared these subgroups, the PWS cases with deletion showed statistically significant lower VIQ scores than that of mUPD [MD=-7.50 (95% CI, -9.75 to -5.26; *p*<0.00001)] and results were homogeneously distributed (*p*=0.71) (Figure 4).

#### PIQ in paternal deletion and mUPD subgroups

**Figure 5:**
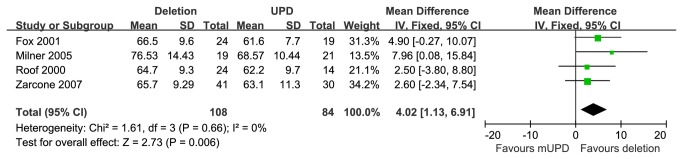
Meta analysis of the PIQ in DEL and UPD groups.

Four studies [21,27,28,32] provided PIQ scores for PWS patients with 15q11-q13 paternal deletion or mUPD. These studies encompassed 108 cases with the paternal deletion and 84 cases with mUPD. Compared with these two subgroups, those with paternal deletion showed statistically significant higher PIQ scores than that of mUPD, [MD=4.02 (95% CI, 1.13 to 6.91; *p*=0.006)] and results were homogeneously distributed (*p*=0.66) (Figure 5).

#### Psychosis in paternal deletion and mUPD subgroups

**Figure 6:**
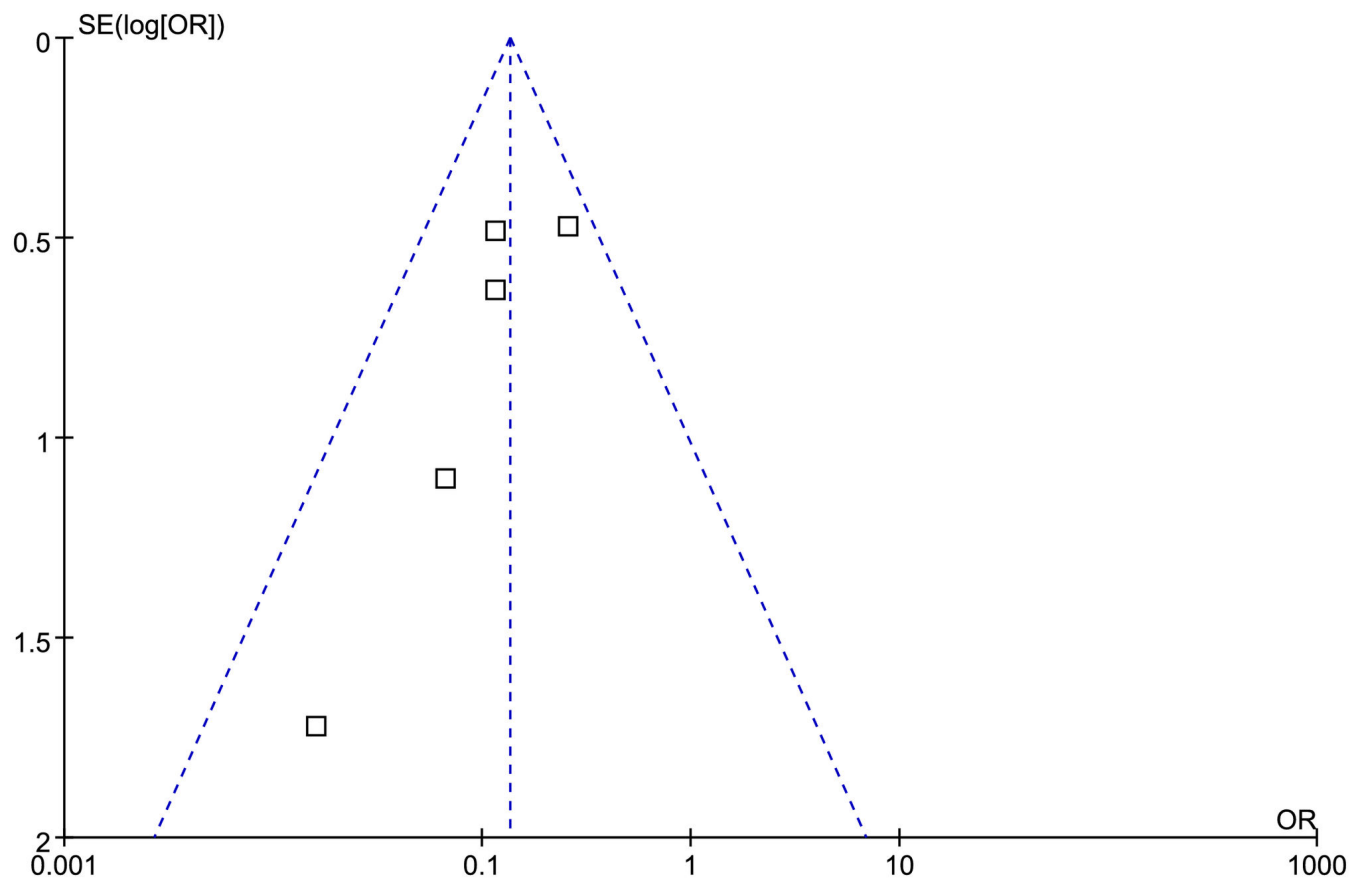
Funnel plot for publication bias about psychosis.

**Figure 7:**
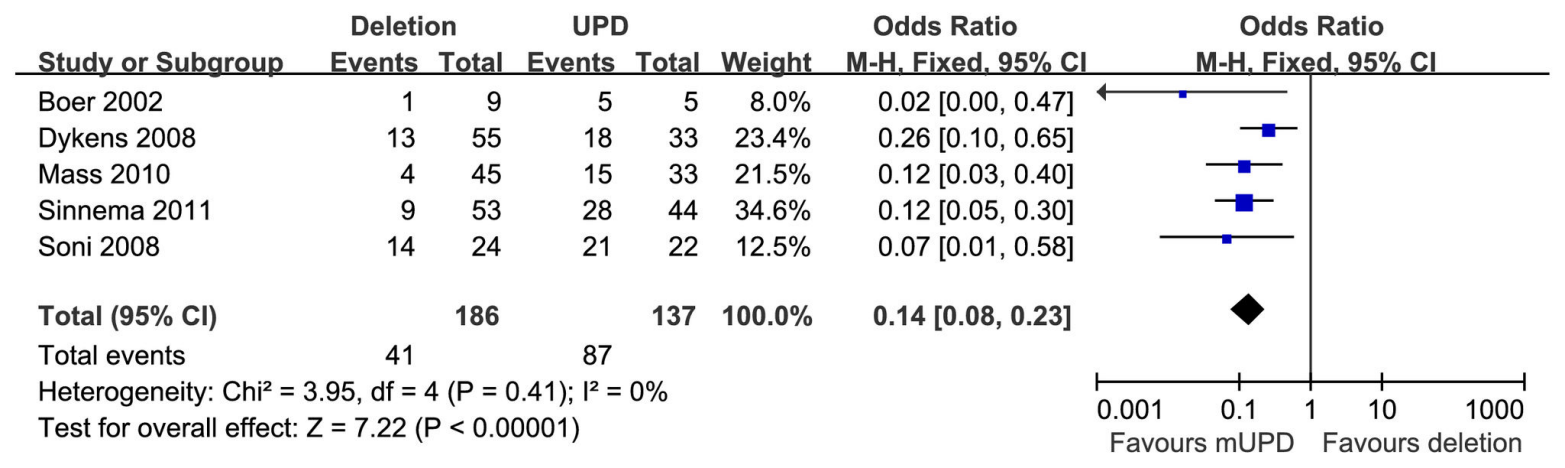
Metaanalysis of the prevalence of psychosis in DEL and UPD groups.

Funnel plot for publication bias about psychiatric illness is presented in Figure 6. Five studies [11,24–26,31] reported prevalence of psychosis in PWS patients with 15q11-q13 paternal deletion or mUPD. These studies encompassed 186 cases with the paternal deletion and 137 cases with mUPD. Compared with the two subtypes, individuals with mUPD were statistically significant more susceptible to psychotic disorder than those with deletion, [OR=0.14 (95% CI, 0.08 to 0.23; *p*<0.00001)] and results were homogeneously distributed (*p*=0.41) (Figure 7).

#### Depressive psychosis in paternal deletion and mUPD subgroups

**Figure 8:**
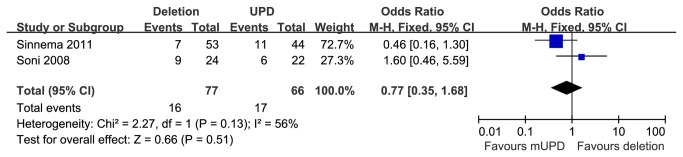
Metaanalysis of the prevalence of depression in DEL and UPD groups.

Two studies [24,26] reported prevalence of depressive psychosis in PWS patients with 15q11-q13 paternal deletion or mUPD. These studies encompassed 77 cases with the paternal deletion and 66 cases with mUPD. The frequency of depressive psychosis between the genetic subtypes were not statistically significant [OR=0.77 (95% CI, 0.35 to 1.68; *p*=0.51)] and results were homogeneously distributed (*p*=0.13) (Figure 8).

#### Bipolar disorder in paternal deletion and mUPD subgroups

**Figure 9:**
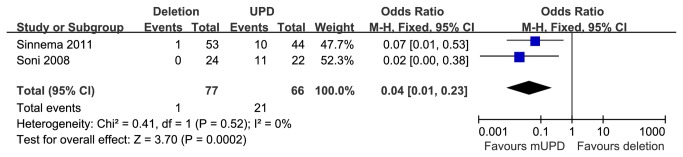
Metaanalysis of the prevalence of bipolar in DEL and UPD groups.

Two studies [24,26] reported prevalence of bipolar illness in PWS patients with 15q11-q13 paternal deletion or mUPD. These studies encompassed 77 cases with the paternal deletion and 66 cases with mUPD. Compared with the two genetic subsets, individuals with mUPD were statistically significant more susceptible to bipolar illness than those with deletion, [OR=0.04 (95% CI, 0.01 to 0.23; *p*=0.0002)] and results were homogeneously distributed (*p*=0.52) (Figure 9).

## Discussion

To our knowledge, this is the first meta-analysis to review the psychiatric illness and intellectual abilities in PWS with different types of molecular defects. Our analysis indicates significant differences of clinical presentation related to the neurodevelopment and psychiatric illness between PWS cases with 15q11-q13 paternal deletion and mUPD. Overall, the PWS with paternal deletion have lower FSIQ score than that of mUPD. More specifically, the verbal IQ but not performance IQ is more affected in the cases with 15q11-q13 deletion. Conversely, the psychosis and bipolar disorders are significantly more prevalent in PWS cases with mUPD than that of 15q11-q13 deletion. These findings not only strengthen the previous impression from small scale studies but also provide new insight that has not reported before.

The immediate question is what contributes to these differences between cases with paternal deletion and mUPD. Human chromosome 15q11–q13 is a domain subject to regulation of genomic imprinting, an epigenetic process in which the expression of genes is depending upon the parent-of-origin [36]. It is well known that PWS primarily arises from the deficiency of paternally expressed gene or genes in the 15q11-q13 region. However, it remains a question of debate which genes or genes are responsible for the full spectrum of PWS phenotypes. Three recent reports from analysis of 3 PWS cases have revealed small but overlapping microdeletions including the *SNORD116* snoRNA cluster [37–39]. These cases have typical clinical features of PWS. However, the neurobehavioral studies have not been evaluated or described in detail. These data support that the paternally expressed *SNORD116* cluster plays a major role in the core features of PWS. However, how the loss of expression non-coding snoRNAs ultimately leads to the clinical presentations of PWS remains unclear. The molecular difference between paternal deletion and mUPD is clear. Summary of the genetic and expression of chromosomal region 15q11, 2-13 are presented in Table 3. In the cases of paternal deletion, the paternally expressed genes in the 15q11-q13 region are predicted to be completely deficient. The maternally expressed genes such as *UBE3A* and *ATP10A* are not affected. The non-imprinted genes in the regions are haploinsufficient in paternal deletion cases. In the mUPD cases, the maternally expressed genes are predicted to be two folds of that in paternal deletion cases and one fold higher than that in normal. The non-imprinted genes are not affected. The expression of imprinted gene is expected to be same as paternal deletion cases if the imprinting is complete or 100%. It is reasonable to hypothesize that the differential expression of genes between paternal deletion of 15q11-q13 and mUPD may contribute to the difference in neurocognitive function and prevalence of psychiatric illness. For instance, the higher expression of *UBE3A* in mUPD cases may play a significant role in psychiatric illness. *UBE3A* is a maternal and brain-specific imprinting gene in the region [40,41]. *UBE3A* encodes ubiquitin protein ligase E3A, functions as an E3 ligase in the ubiquitin proteasome pathway and as a transcriptional coactivator [42]. Loss of function of maternal *UBE3A* causes Angelman syndrome, a neurodevelopmental disorder with intellectual disability, hypotonia and seizures [43,44]. The maternal duplication of 15q11-q13 is one of the most common copy number variants found in autism spectrum disorder [45]. It has been suggested that the *UBE3A* may be the major player to contribute the ASD in these cases [46,47]. Other gene such as *CYFIP1* in the 15q11.2 region may also play a role in contributing to neurodevelopment and psychiatric illness because of copy number variant of 15q11.2 including *CYFIP1* has been associated with schizophrenia [48]. *CYFIP1* encodes cytoplasmic *FMR1* interacting protein 1, and component of the CYFIP1**-**EIF4E-FMR1 complex which binds to the mRNA cap and mediates translational repression [49].

**Table 3:** Summary of the genetic and expression of chromosomal region 15q11, 2-13.

**Gene name **	**Description**	**Imprinting status**	**Expression level (Deletion) **	**Expression level (mUPD) **	**Comparison in two subtypes**
TUBGCP5	tubulin, gamma complex associated protein 5	non-imprinted	haploinsufficiency	normal	different
CYFIP1	cytoplasmic FMR1 interacting protein 1	non-imprinted	haploinsufficiency	normal	different
NIPA2	non imprinted in Prader-Willi/Angelman syndrome 2	non-imprinted	haploinsufficiency	normal	different
NIPA1	non imprinted in Prader-Willi/Angelman syndrome 1	non-imprinted	haploinsufficiency	normal	different
GOLGA8E	golgin A8 family, member E, pseudogene	non-imprinted	haploinsufficiency	normal	different
MKRN3	makorin ring finger protein 3	paternally expressed	no expression	no expression	same
MAGEL2	MAGE-like 2	paternally expressed	no expression	no expression	same
NDN	necdin, melanoma antigen (MAGE) family member	paternally expressed	no expression	no expression	same
NPAP1	nuclear pore associated protein 1	non-imprinted	haploinsufficiency	normal	different
SNRPN	small nuclear ribonucleoprotein polypeptide N	paternally expressed	no expression	no expression	same
SNURF	SNRPN upstream reading frame	paternally expressed	no expression	no expression	same
snoRNAs	small nucleolar RNA	paternally expressed	no expression	no expression	same
UBE3A	ubiquitin protein ligase E3A	maternally expressed	normal	over expression	different
ATP10A	ATPase, class V, type 10A	maternally expressed	normal	over expression	different
GABRB3	gamma-aminobutyric acid (GABA) A receptor, beta 3	paternal biased expression	haploinsufficiency	haploinsufficiency	same
GABRA5	gamma-aminobutyric acid (GABA) A receptor, alpha 5	paternal biased expression	haploinsufficiency	haploinsufficiency	same
GABRG3	gamma-aminobutyric acid (GABA) A receptor, gamma 3	non-imprinted	haploinsufficiency	normal	different
OCA2	oculocutaneous albinism II	non-imprinted	haploinsufficiency	normal	different
GOLGA8G	golgin A8 family, member G	non-imprinted	haploinsufficiency	normal	different

The clinical implication of our finding is significant because it provides a basis to develop evidence based practice guideline for evaluating and managing individuals with PWS. In particular, our findings provide evidence to support more diligent care of PWS with psychiatric illness.

Our conclusion from the meta-analysis is convincing and significant. However, several imitations in our study may still be worth for discussion and for improvement in future. First, like all other meta-analysis, because clinic data were collected from different investigators, at different sites, and at different ages, the comparison may be confounded by the variable quality of clinical data. Second, it should be noted that the diagnosis of psychiatric diseases in many reports included in meta-analysis might be made based on self-report but not through a specialized clinical evaluation using DSM IV criteria. These limitations may influence the data quality thereby affect the conclusion. Therefore, more accurate and detailed description about psychiatric symptoms is recommended for further study of PWS to figure out underlying molecular mechanism. Third, although the total number of patients is reasonable, the increase of the sample size may still be desirable in future study. Fourth, the breakpoints for the deletion case are not defined in detail.

## Conclusion

Our meta-analysis has revealed significant differences in the severity of neurocognitive impairment and the prevalence of psychiatric illness between PWS cases with paternal deletion of 15q11-q13 and mUPD. The PWS with paternal deletion have lower FSIQ and VIQ score than that of mUPD, but PWS with mUPD are more susceptible to psychosis, bipolar disorders than that of paternal deletion. These findings may provide a framework for future study to elucidate the genetic basis of psychiatric illness. Our finding will also provide rationale to develop evidence based practice for evaluating and caring individuals with PWS.

## Supporting Information

Table S1 **PRISMA 2009 Checklist.**
(DOC)
